# Potential predictive biomarkers in antitumor immunotherapy: navigating the future of antitumor treatment and immune checkpoint inhibitor efficacy

**DOI:** 10.3389/fonc.2024.1483454

**Published:** 2024-11-25

**Authors:** Xiangyu Yin, Yunjie Song, Wanglong Deng, Neil Blake, Xinghong Luo, Jia Meng

**Affiliations:** ^1^ Department of Biological Sciences, School of Science, AI University Research Centre, Xi’an Jiaotong-Liverpool University, Suzhou, China; ^2^ Institute of Biomedical Research, Regulatory Mechanism and Targeted Therapy for Liver Cancer Shiyan Key Laboratory, Hubei Provincial Clinical Research Center for Precise Diagnosis and Treatment of Liver Cancer, Taihe Hospital, Hubei University of Medicine, Shiyan, Hubei, China; ^3^ Institute of Infection, Veterinary & Ecological Sciences, University of Liverpool, Liverpool, United Kingdom; ^4^ Jiangsu Simcere Diagnostics Co., Ltd., The State Key Laboratory of Neurology and Oncology Drug Development, Nanjing, China; ^5^ Institute of Systems, Molecular and Integrative Biology, University of Liverpool, Liverpool, United Kingdom

**Keywords:** immunotherapy, immune checkpoint inhibitors, PD-L1, TMB, MSI, emerging biomarkers

## Abstract

Immune checkpoint inhibitors (ICIs) have revolutionized cancer treatment modality, offering promising outcomes for various malignancies. However, the efficacy of ICIs varies among patients, highlighting the essential need of accurate predictive biomarkers. This review synthesizes the current understanding of biomarkers for ICI therapy, and discusses the clinical utility and limitations of these biomarkers in predicting treatment outcomes. It discusses three US Food and Drug Administration (FDA)-approved biomarkers, programmed cell death ligand 1 (PD-L1) expression, tumor mutational burden (TMB), and microsatellite instability (MSI), and explores other potential biomarkers, including tumor immune microenvironment (TIME)-related signatures, human leukocyte antigen (HLA) diversity, non-invasive biomarkers such as circulating tumor DNA (ctDNA), and combination biomarker strategies. The review also addresses multivariable predictive models integrating multiple features of patients, tumors, and TIME, which could be a promising approach to enhance predictive accuracy. The existing challenges are also pointed out, such as the tumor heterogeneity, the inconstant nature of TIME, nonuniformed thresholds and standardization approaches. The review concludes by emphasizing the importance of biomarker research in realizing the potential of personalized immunotherapy, with the goal of improving patient selection, treatment strategies, and overall outcomes in cancer treatment.

## 1 Introduction

Immunotherapy, especially immune checkpoint inhibitors (ICIs), has revolutionized the landscape of cancer treatment (1). Compared to conventional treatment strategies such as radiotherapy, chemotherapy, and targeted drugs, ICIs exhibited superior efficacy for certain types of cancer, particularly in tumors with progression or recurrence (2). Immune checkpoints encompass a series of immunosuppressive molecules, represented by programmed cell death protein 1 (PD-1), programmed cell death ligand 1 (PD-L1), and cytotoxic-T-lymphocyte-associated protein 4 (CTLA-4), which primarily function to maintain self-immune tolerance and suppress excessive auto-immunity (3, 4). By targeting this mechanism, tumor cells can activate the immune checkpoints to prevent T-cell activation, thereby evading the surveillance of the immune system (5). ICIs work by blocking these inhibitory signals to promote the tumor immune response and inhibit tumor growth.

There are several immunosuppressive pathways in the activation of T cells and the interaction of activated T cells with tumor cells, and existing immune checkpoint blockades target two specific pathways, CTLA-4/B7-1/2 and PD-1/PD-L1 (6). CTLA-4/B7-1/2 pathway functions in regulating the early-stage T cell activation in lymph nodes (7). CTLA-4 molecule is expressed by CD4+ T cells and CD8+ T cells, and it binds with its ligand B7-1/2 (CD80/CD86) to suppress T cell activation signals, thereby blocking the antitumor process (8, 9). Targeting on the CTLA-4 pathway, ipilimumab is the first developed antibody approved by the US Food and Drug Administration (FDA). The PD-1/PD-L1 pathway is the most thoroughly investigated and has led to the development of several FDA-approved drugs, including PD-1 inhibitors like pembrolizumab, nivolumab, cemiplimab, as well as PD-L1 inhibitors, such as atezolizumab, and durvalumab. PD-1/PD-L1 pathway functions in the later stage of immune response, which primarily limit the proliferation, differentiation and activation of T cells (7). PD-1 (CD279) is expressed by various immune cells, including activated T cells, natural killer cells, B cells, macrophages, monocytes, and dendritic cells (DCs), and its ligand PD-L1/2 is predominantly expressed on tumor cells and can also be found on the surface of activated T cells, B cells, epithelial cells, and DCs (10–13). These pathways inhibit the activation and proliferation of T leukocytes, induce apoptosis in activated T leukocytes, and enhance the immune evasion of tumor cells (4). Hence, understanding these intricate interactions within immune cells can lay the foundation for further novel developments in immunotherapy.

ICIs have yielded satisfactory results in improving overall survival (OS) rates in various types of tumors, such as melanoma and non-small cell lung cancer (NSCLC) (14–16). However, fewer than half of patients benefit from ICI therapy, and some may suffer from immune-related adverse events (17–19). This emphasizes the importance of patient stratification, to enable personalized and effective treatment for each patient. Given this, the role of biomarkers becomes critical. Biomarkers can facilitate more accurate patient stratification, ensuring that each patient receives appropriate and effective treatment. Thus, exploring and validating effective biomarkers for predicting treatment response and monitoring possible side effects could optimize therapeutic strategies and improve clinical outcomes for patients undergoing ICI treatments.

**Figure 1:**
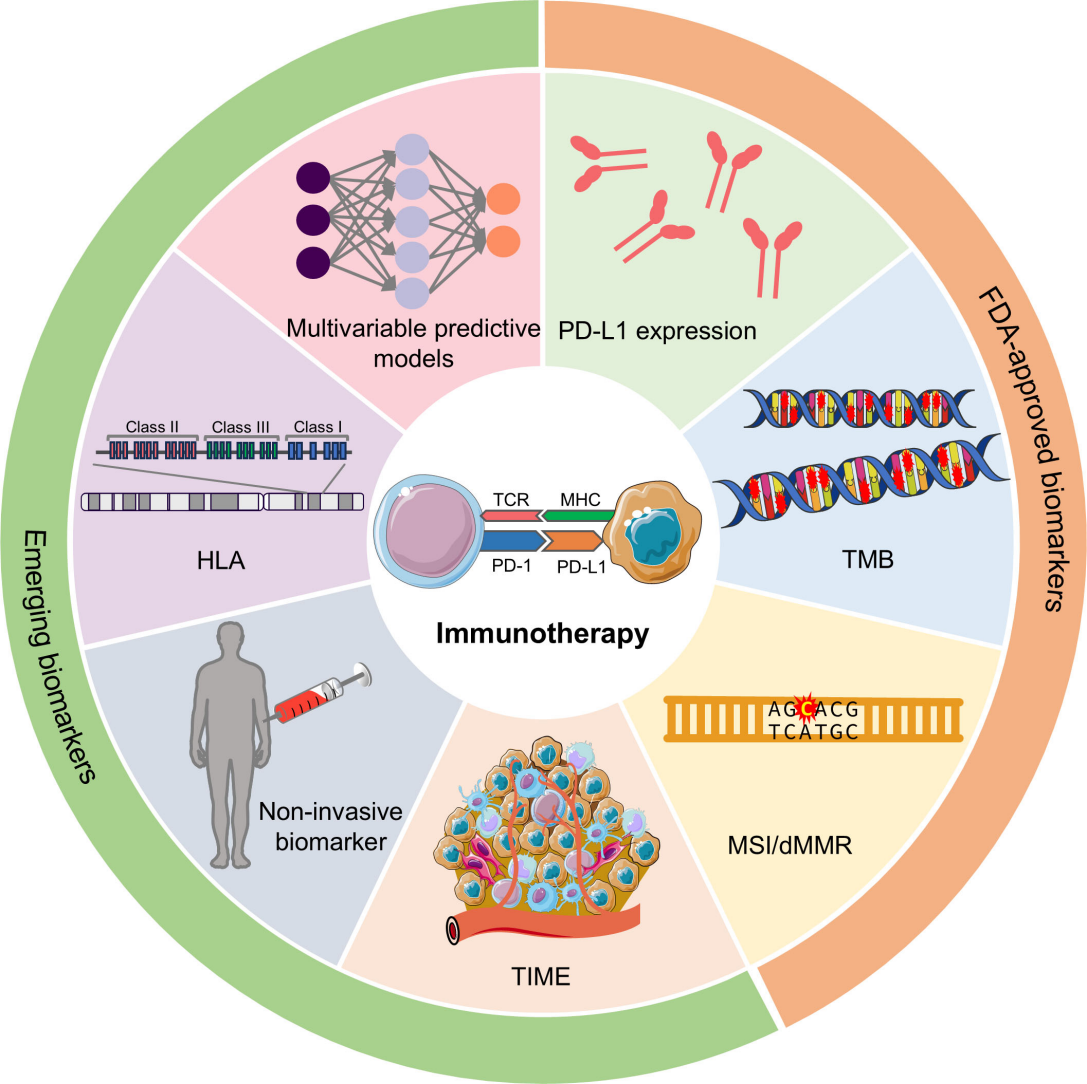
Potential predictive biomarkers in predicting the response to immunotherapy. According to the clinical utility, biomarkers can be divided into FDA-approved biomarkers and emerging biomarkers. FDA-approved biomarkers include PD-L1 expression, TMB, and MSI/dMMR. Emerging biomarkers have many categories, and the most representative ones are TIME-related signatures, non-invasive biomarkers, HLA, and multivariable predictive models. PD-L1, programmed cell death ligand 1; TMB, tumor mutational burden; MSI, microsatellite instability; dMMR, deficiency of DNA mismatch repair; TIME, tumor immune microenvironment; HLA, human leukocyte antigen.

As of yet, there are limited reliable biomarkers to predict the efficacy of ICIs. Only three such biomarkers have garnered FDA approval and are widely used in clinical applications: PD-L1 expression, tumor mutational burden (TMB), and microsatellite instability/deficiency of DNA mismatch repair (MSI/dMMR) (20). This review will provide an overview of these widely used biomarkers, explore several promising biomarkers, such as tumor immune microenvironment (TIME)-related signatures, human leukocyte antigen (HLA), non-invasive biomarkers and others (such as gut microbiome, psychological biomarker, and image-derived biomarker), discuss multivariable models in terms of their performance in predicting the response to ICI-based immunotherapy (**Figure 1**), and identify their limitations. Additionally, we will also outline future research directions aimed at developing more precise and patient-responsive biomarkers. The ultimate goal is to find biomarkers that can accurately predict immunotherapy outcomes, leading to more effectively personalized treatment strategies.

## 2 FDA-approved biomarkers

### 2.1 PD-L1 expression

PD-L1 expression, typically accessed by immunohistochemistry, is one of the most commonly used biomarkers for immunotherapy response prediction. The over-expression of PD-L1 by tumor cells effectively suppresses the immune response and enables the tumor cells to evade the detection, recognition, and destruction of immune cells. Moreover, the quantification of PD-L1 expression primarily relies on two scoring methods: the tumor proportion score (TPS, proportion of stained tumor cells compared to all tumor cells of the sample) and the combined positive score (CPS, the ratio of PD-L1 stained tumor cells to all tumor cells multiplied by 100) (21, 22).

PD-L1 expression has been identified as a significant independent predictive biomarker of ICIs effectiveness across multiple cancer types, such as breast cancer, NSCLC, urothelial carcinoma, and hepatocellular carcinoma (23–25). **Table 1** summarizes the clinical trials of PD-L1 as an independent immunotherapy biomarker in recent years. In the KEYNOTE028 trial, PD-L1 CPS was significantly associated with prolonged progression-free survival (PFS) in patients treated with pembrolizumab (p = 0.005) (18). Similarly, the phase 3 nivolumab CheckMate 459 trial showed a trend towards a higher objective response rate (ORR) in patients with advanced hepatocellular carcinoma who had high PD-L1 expression level (TPS ≥ 1%), albeit not statistically significant (ORR 28% vs 12%) (26).

**Table 1:** Clinical trials of PD-L1 to predict immunotherapy in recent years.

Trial	Cancer type	Number of patients	Immunotherapy strategy	Clinical end points	Main results	Reference
CheckMate 067 (NCT01844505) phase 3	Advanced melanoma	945	Nivolumab + ipilimumab or nivolumab monotherapy or ipilimumab monotherapy	PFS, OS	Patients with both PD-L1 high and PD-L1 low expression showed better ORR, PFS, and OS in nivolumab + ipilimumab and nivolumab monotherapy than ipilimumab monotherapy.	PMID: 31562797 (14)
KEYNOTE-028 (NCT02054806) phase Ib	20 types of advanced solid tumors	475	Pembrolizumab	ORR, PFS, OS	PD-L1 CPS was significantly associated with both higher ORR (p = 0.018), and prolonged PFS (p = 0.005).	PMID: 30557521 (18)
CheckMate 459 (NCT02576509) phase 3	Advanced hepatocellular carcinoma	743	Nivolumab or sorafenib	PFS, OS	Nivolumab-treated patients with high PD-L1 expression level (TPS ≥ 1%) tend to have a higher ORR than the low-level patients (28% vs 12%).	PMID: 34914889 (26)
KEYNOTE-024 (NCT02142738) phrase 3	Advanced non-small cell lung cancer	305	Pembrolizumab or chemotherapy	ORR, PFS, OS	Pembrolizumab was significantly associated with longer PFS (p < 0.001) and OS (p = 0.005) in patients with PD-L1 TPS ≥ 50%.	PMID: 27718847 (118)
CheckMate 040 (NCT01658878) phase 1/2	Advanced hepatocellular carcinoma	262	Nivolumab	ORR, PFS, OS	Patients with tumor cell PD-L1 expression ≥ 1% had significantly improved OS (p = 0.032).	PMID: 32710922 (119)
IMpassion130 (NCT02425891) phase 3	Advanced triple-negative breast cancer	902	Atezolizumab + nab-paclitaxel or placebo + nab-paclitaxel	PFS, OS	Median PFS was significantly prolonged in PD-L1 TPS ≥ 1% patients treated with atezolizumab compared with placebo (p < 0.001). Median OS was about 25 vs 15.5 months.	PMID: 30345906 (120)
CheckMate 649 (NCT02872116) phase 3	Gastric, gastro-oesophageal junction, and oesophageal adenocarcinoma	1581	Nivolumab + chemotherapy or chemotherapy monotherapy	PFS, OS	Patients with PD-L1 CPS ≥ 5 had both prolonged PFS and OS (both p < 0.0001). Patients with PD-L1 CPS ≥ 1 also had prolonged OS (p < 0.0001).	PMID: 34102137 (121)
CheckMate 227 (NCT02477826) phase 3	Advanced non-small cell lung cancer	1739	Nivolumab + ipilimumab or chemotherapy monotherapy	PFS, OS	Nivolumab + ipilimumab prolonged OS of patients compared with chemotherapy, regardless of PD-L1 expression level.	PMID: 31562796 (122)
CheckMate 274 (NCT02632409) phase 3	Muscle-invasive urothelial carcinoma	709	Nivolumab or placebo	PFS	Among patients with PD-L1 expression ≥ 1%, the 6-month DFS was significantly higher in patients treated with nivolumab compared with placebo (p < 0.001).	PMID: 34077643 (123)
IMpower150 (NCT02366143) phase 3	Metastatic nonsquamous non-small cell lung cancer	1202	Atezolizumab-carboplatin-paclitaxel (ACP), atezolizumab-bevacizumab-carboplatin-paclitaxel (ABCP), bevacizumab-carboplatin-paclitaxel (BCP)	PFS, OS	Longer PFS and OS were observed among patients treated with ABCP and ACP compared with BCP in both PD-L1 positive and PD-L1 high subgroups.	PMID: 34311108 (124)

PD-L1, programmed cell death ligand 1; ORR, objective response rate; PFS, progression-free survival; OS, overall survival; TPS, tumor proportion score; CPS, combined positive score.

However, conflicting results were observed in other studies. A retrospective study of NSCLC patients exhibited worse PFS in patients with positive PD-L1 level (TPS ≥ 1%) (27). In CheckMate 067 trial of advanced melanoma, patients with both high and low expression level of PD-L1 showed better ORR, PFS, and OS in nivolumab-plus-ipilimumab combination and nivolumab monotherapy than ipilimumab alone (27, 28). Similar results were found in NSCLC patients in CheckMate 017 and CheckMate 057 trials (29, 30). It can be concluded from these studies that PD-L1 may not be an eligible predictive biomarker in certain patient populations.

Despite its usefulness, there are some limitations for PD-L1 expression as an independent biomarker, which may influence its predictive accuracy (31). These include variations in detection platforms and assays, especially the specific diagnostic antibody in immunohistochemistry and the PD-L1 staining method; the discrepancies of quality for tumor specimens and the approach for sampling gathering; different threshold values used to define PD-L1 positivity; and the spatial and temporal heterogeneity of PD-L1 expression (32–35). All these factors contribute to conflicting data regarding immunotherapy efficacy on PD-L1 expression across various studies. Besides, it is noticed that PD-L1 status is not constant but changes dynamically during the treatment. Thus, it is necessary to address this issue utilizing dynamic detection (23).

### 2.2 Tumor mutational burden

TMB is defined as the number of non-synonymous somatic mutations occurring per megabase (muts/Mb). Theoretically, TMB is accurately gauged through whole-exome sequencing, but big-panel-based sequencing is commonly adopted in clinical trials (36). The definition of TMB encompasses three types of mutations: all mutations, non-synonymous mutations, and mutations in protein-coding regions (37). The primary challenge in TMB assessment is the need for unification in sequencing panels and mutation types employed across studies.

TMB is reported as a significant biomarker that can discriminate patients responsive to ICIs in numerous studies (**Table 2**). Patients displaying high TMB levels (TMB-H) usually respond better to ICI therapy. An elevated TMB level correlates with more mutations, generating more neo-antigens, which consequently amplify the recognition by the immune cells. These neo-antigens serve as tumor-specific targets, and ICI-based immunotherapy can strengthen the antitumor response of T cells by recognizing and eliminating tumor cells carrying a high neo-antigen burden.

**Table 2:** Clinical trials of TMB to predict immunotherapy in recent years.

Trial	Cancer type	Number of patients	Immunotherapy strategy	Clinical end points	Main results	Reference
KEYNOTE-028 (NCT02054806) phase Ib	20 types of advanced solid tumors	475	Pembrolizumab	ORR, PFS, OS	Higher TMB was significantly associated with patients who achieved ORR (p = 0.018) and had longer PFS (p = 0.051).	PMID: 30557521 (18)
CheckMate 275 (NCT02387996) phase 2	Metastatic urothelial carcinoma	270	Nivolumab	ORR, PFS, OS	Higher TMB was significantly associated with improved ORR, PFS, and OS (all p < 0.05).	PMID: 32532789 (24)
KEYNOTE-158 (NCT02628067) phase 2	10 types of advanced solid tumors	1066	Pembrolizumab	ORR, PFS, OS	TMB-H (> 10 muts/Mb) patients had larger ORR than non-TMB-H patients (29% vs 6%).	PMID: 32919526 (38)
KM-06 (NCT04761744) phase 2	Refractory solid cancers	48	Nivolumab	ORR, PFS, OS	Patients with TMB-H (TMB > 12 muts/Mb) had significantly longer median PFS (p = 0.0061).	PMID: 38485184 (40)
CheckMate 568 (NCT02659059) phase 2	Advanced non-small cell lung cancer	288	Nivolumab + ipilimumab	ORR, PFS	Patients with high TMB (≥ 10 muts/Mb) had higher ORR than those with low TMB (< 10 muts/Mb).	PMID: 30785829 (114)
CheckMate 227 (NCT02477826) phase 3	Advanced non-small cell lung cancer	1739	Nivolumab + ipilimumab or nivolumab monotherapy or chemotherapy monotherapy	PFS, OS	Patients with TMB-H (≥ 10 muts/Mb) had significantly longer PFS when treated with nivolumab + ipilimumab than chemotherapy (p = 0.007).	PMID: 29658845 (125)
CheckMate 026 (NCT02041533) phase 3	Stage IV or recurrent non-small cell lung cancer	541	Nivolumab or chemotherapy	PFS, OS	Patients with a high TMB had higher response rate and longer PFS with nivolumab than chemotherapy.	PMID: 28636851 (113)
KEYNOTE-086 (NCT02447003) phase 2	Metastatic triple-negative breast cancer	254	Pembrolizumab	ORR, PFS, OS	TMB was significantly associated with improved ORR (p = 0.007), PFS (p = 0.034), and OS (p = 0.025).	PMID: 37099733 (126)
KEYNOTE-062 (NCT02494583) phase 3	Advanced gastric cancer	763	Pembrolizumab + chemotherapy or pembrolizumab monotherapy or chemotherapy monotherapy	ORR, PFS, OS	TMB (≥ 10 muts/Mb) was significantly associated with ORR, PFS, and OS in patients treated with pembrolizumab + chemotherapy, and pembrolizumab (all p < 0.05).	PMID: 35657979 (127)

TMB, tumor mutational burden; ORR, objective response rate; PFS, progression-free survival; OS, overall survival.

The KEYNOTE-028 study of 20 cancer types indicated that participants who achieved ORR and extended PFS had significantly higher TMB (p = 0.018 and p = 0.051, respectively) (18). As reported in the phase 2 clinical trial KEYNOTE-158, 29% of TMB-H (> 10 muts/Mb) patients and 6% of low TMB level (TMB-L) patients showed response to ICI therapy (38). A study on advanced cutaneous melanoma patients found that TMB value was significantly higher in responders compared with non-responders, and higher TMB was associated with improved PFS (p < 0.0001) (39). The KM-06 trial of refractory solid tumors used a threshold value of 12 muts/Mb, and the patients with TMB-H (TMB > 12 muts/Mb) had significantly longer median PFS on nivolumab responses (p = 0.0061) (40).

However, due to intra- and inter-tumoral heterogeneity, TMB level varies among different sites and different cancer types. Kazdal et al. (2019) studied the TMB heterogeneity between the primary site and the lymph node metastases, and 24 samples of lung adenocarcinoma was enrolled for paired sampling. It was noted that significantly lower TMB was observed in lymph node compared to the primary site (p = 0.016) (41). Besides, TMB level was proved to have high variance among different cancer types. Over 100,000 cancer samples of more than 100 cancer types in a study had a median TMB value of 3.6 muts/Mb, but the range of median TMB was from 0.8 muts/Mb in bone marrow myelodysplastic syndrome to 45.2 muts/Mb in skin squamous cell carcinoma (36). Moreover, the cut-off values of defining TMB-H are even different in same cancer type among different studies. For studies on NSCLC, Meng et al. (2022) defined TMB-H to be ≥16 muts/Mb (42), while the study MYSTIC used a threshold TMB value of ≥10 muts/Mb (43).

Due to the heterogeneity among differences in regions of the same tumor, cancer types, detection panels, accessed tissues, and calculation methods, it is tough to establish a universal threshold value for defining a high TMB level. Founder effect can also lead to differences in TMB between different population of patients. For instance, patients with germline gene BRCA1 or BRCA2 mutations had significantly higher TMB (p = 0.004) than the non-carriers (44). Moreover, TMB-H is not a perfect predictive biomarker, as some TMB-L patients also respond well to immunotherapy (45, 46). Future research should aim to construct a uniform diagnostic standard of TMB-H, especially in standardizing the TMB estimates across various platforms or panels. Despite the challenge of reconciling variations across cancer types, establishing specific cutoff values for particular cancer types could serve as a useful reference.

### 2.3 Microsatellite instability

MSI is characterized by genetic alterations in the length of microsatellite DNA sequences (47). Microsatellites, also known as short tandem repeats, are repeating sequences of 1-6 base pairs of DNA that are dispersed throughout the genome. MSI is the result of deficiency of DNA mismatch repair (dMMR), usually caused by the mutation of MMR-related genes, which are *MSH2*, *MSH6*, *MLH1*, and *PMS2* (48, 49). The deficiency of MMR function attenuates the capability of correcting the errors occurred during DNA replication and maintaining the integrity of the genome. It would result in the accumulation of genetic mutations, particularly in microsatellite regions, thus escalating the risk of tumorigenesis.

MSI is observed in various types of cancers, including endometrial, colorectal, gastric, pancreatic, brain, biliary tract, urinary tract, and ovarian tumors, among which endometrial, colorectal, and gastric cancers are the most prevalent ones (48, 50). MSI status can be categorized based on its degree into microsatellite instability high (MSI-H), microsatellite instability low (MSI-L), and microsatellite stability (MSS). However, there has always been debate about the threshold of defining MSI-H, with variations across different detection methods. The gold standard detection method of MSI is the fluorescence multiplex polymerase chain reaction and capillary electrophoresis, but NGS-based panels have gradually become prevalent in MSI detection (47). MSI-H/dMMR has been proven to symbolize responsiveness to immunotherapy in several cancers (**Table 3**).

**Table 3:** Clinical trials of MSI status to predict immunotherapy in recent years.

Trial	Cancer type	Number of patients	Immunotherapy strategy	Clinical end points	Main results	Reference
KEYNOTE-016 (NCT01876511) phrase 2	Colorectal cancer (CRC) and non-CRC cancer	41	Pembrolizumab	Immune-related ORR and the 20-week immune-related PFS rate	ORR was 40% in the dMMR CRC cohort, and 0% in the pMMR CRC cohort. The 20-week PFS was 78% in dMMR CRC cohort, compared with 11% in the pMMR CRC cohort.	PMID: 26028255 (51)
KEYNOTE-177 (NCT02563002) phase 3	Metastasis colorectal cancer	307	Pembrolizumab or chemotherapy	PFS, OS	MSI-H patients treated with pembrolizumab exhibited significantly longer PFS than those treated with chemotherapy (p = 0.0002).	PMID: 33264544 (52)
NCT03981796 phase 3	Primary advanced or recurrent endometrial cancer	494	Dostarlimab + carboplatin-paclitaxel or placebo + carboplatin-paclitaxel	PFS, OS	In patients with dMMR/MSI-H, PFS was significantly longer with dostarlimab than placebo (61.4% vs 15.7%, p < 0.001).	PMID: 36972026 (128)
Study 309-KEYNOTE-775 (NCT03517449) phase 3	Advanced endometrial cancer	827	Lenvatinib + pembrolizumab or chemotherapy monotherapy	PFS, OS	Patients with pMMR had significantly longer PFS and OS with lenvatinib + pembrolizumab than chemotherapy (both p < 0.001).	PMID: 35045221 (129)
NRG-GY018 (NCT03914612) phase 3	Advanced or recurrent endometrial cancer	816	Paclitaxel-carboplatin + pembrolizumab or paclitaxel-carboplatin + placebo	PFS, OS	In the dMMR cohort, PFS was significantly longer in patients treated with pembrolizumab than those with placebo (p < 0.001).	PMID: 36972022 (130)
GARNET (NCT02715284) phase 1	Endometrial cancer	290	Dostarlimab	ORR, DOR	ORR in dMMR/MSI-H patients was much higher than pMMR/MSS patients (43.5% vs 14.1%).	PMID: 35064011 (131)

MSI, microsatellite instability; MSS, microsatellite stability; dMMR, deficiency of DNA mismatch repair; pMMR, proficiency of DNA mismatch repair; ORR, objective response rate; PFS, progression-free survival; OS, overall survival; DOR, duration of response.

In the KEYNOTE-016 trial of metastatic colorectal cancer (CRC), pembrolizumab monotherapy-treated patients were categorized into three cohorts according to MMR status and cancer type (51). The ORR was 40% in the dMMR CRC cohort, 0% in the proficient-MMR (pMMR) CRC cohort, and 71% in the dMMR non-CRC cohort. It can be inferred that CRC patients with dMMR/MSI-H are more likely to respond positively to immunotherapy. The KEYNOTE-117 trial enrolled metastatic CRC patients and found that patients with MSI-H/dMMR had significantly better PFS with pembrolizumab therapy compared to the standard chemotherapy (hazard ratio (HR) = 0.60, 95% confidence interval (CI) 0.45-0.80, p = 0.002) (52). A meta-analysis integrated patients diagnosed with advanced gastric cancer from four phase 3 trials (KEYNOTE-062, CheckMate 649, JAVELIN Gastric 100, and KEYNOTE-061) was performed to explore the predictive power of MSI on immunotherapy (53). Patients with MSI-H demonstrated significantly lower HR than the MSS patients on OS (HR 0.34 vs 0.85, p = 0.003). Meanwhile, MSI-H patients also had significantly better PFS (p = 0.04) and a higher response rate to treatment (p = 0.02).

Despite the enduring clinical benefits conferred by ICI-based therapy, its efficacy is predominantly confined to a selected subset of patients harboring tumors of MSI-H status. Furthermore, patients who initially exhibit a favorable response may ultimately develop resistance to therapy (54).

## 3 Emerging biomarkers

### 3.1 Tumor immune microenvironment-related signatures

The characteristics of TIME are proven to be associated with the efficacy of immunotherapy of immune checkpoint blockades. Gene expression profiling (GEP) signatures within the TIME, such as tumor inflammation signature (TIS) (55), epithelial-to-mesenchymal transition (EMT) signatures (30), and innate anti-PD-1 resistance (IPRES) (56) and the level of tumor-infiltrating lymphocytes (TILs) (57) can serve as indicators for immunotherapy outcomes. These signatures are primarily accessed through next-generation sequencing techniques like RNA-seq, which reveal specific GEP patterns of the TIME using small gene clusters, also referred to as gene expression signatures.

#### 3.1.1 Tumor inflammation signature

The 18-gene tumor inflammation signature (TIS) signature, composed of genes related to antigen presentation, IFN-gamma activity, and immune cell function, is calculated by the weighted sum expression of 18 normalized marker genes (58). TIS, initially developed to predict ICI response in melanoma, has shown a significant correlation with treatment outcomes (59). The CRETIM pan-cancer cohort validated a strong correlation between higher TIS scores and improved response rates, including complete response and partial response in patients treated with nivolumab and pembrolizumab (odd ratio (OR) = 2.64, 95% CI 1.4-6.0, p = 0.008). Additionally, patients with higher TIS scores also exhibited prolonged overall survival (HR = 0.37, 95% CI 0.18-0.76, p = 0.005) (55). These findings were further corroborated by the KEYNOTE-028 trial, which demonstrated a significant association between TIS and ORR (p = 0.012) and PFS (p = 0.017) in a cohort encompassing up to 20 different cancer types (18).

#### 3.1.2 Epithelial-to-mesenchymal transition signature

The 12-gene signature epithelial-to-mesenchymal transition (EMT) indicates the immunosuppressive intensity of tumor cells, potentially leading to resistance to anti-PD-1 therapy (30). The EMT score is derived by subtracting the summed log2 Z-scores of epithelial genes from the sum of the log2 Z-scores of the mesenchymal genes. For instance, in NSCLC, responders had significantly lower (more epithelial genes) EMT signature scores as opposed to non-responders (p = 0.016) (30). Similarly, another study of advanced malignant peritoneal mesothelioma yielded consistent results, spotting a significant correlation between a high EMT score and poor ORR (60).

#### 3.1.3 Innate anti-PD-1 resistance

The 26-gene signature known as innate anti-PD-1 resistance (IPRES) highlights the up-regulation of several biological processes, such as mesenchymal transition, cell adhesion, and angiogenesis, contributing to immune suppression during anti-PD-1 therapy (56). In the melanoma cohort, significant overexpression of IPRES was noted among patients non-responsive to anti-PD-1 therapy (OR = 4.6, p = 0.013), while under-enrichment was observed in responders (OR = 0.15, p = 0.04) (56). This suggests that targeting IPRES-related pathways could potentially ameliorate anti-PD-1 responses. However, similar results were not consistently replicated in other cohorts, hinting at the possibility of IPRES being a cohort-specific signature (61).

#### 3.1.4 Tumor-infiltrating lymphocytes

TILs refer to the infiltration of lymphocytes in tumor tissues, typically CD8+ T cells and CD4+ T cells (62). Originating from the migration of circulating blood lymphocytes, the intensity of TILs infiltration of the tumor or the tumor stroma indicates tumor status (63–65). The abundance and functional status of TILs have been implicated in ICI-based immunotherapy response. A landmark study showed that in advanced melanoma patients treated with pembrolizumab, higher levels of TILs, particularly CD8+ T cells, were correlated with improved response and survival rates (57). A retrospective study on advanced NSCLC highlighted significant correlations between TILs and PFS (HR = 0.50, 95% CI 0.34-0.74, p < 0.001) as well as OS (HR = 0.37, 95% CI 0.24-0.57, p < 0.001) (66). However, the composition of TILs is crucial. For instance, a predominance of regulatory T cells (Treg) within the TILs can serve as an antitumoral effector with a poorer prognosis (67, 68).

The TIME plays a critical role in determining the efficacy of ICIs. Various gene expression signatures and the presence of TILs serve as important biomarkers for predicting patient responses to these therapies. In addition to the above scientific findings, there are also ongoing clinical trials conducted to validate the effectiveness of TIME-related signatures, especially TILs, as predictive biomarkers of cancer immunotherapy (**Table 4**). While promising, the variability in assessment methods and patient heterogeneity pose challenges in standardizing these biomarkers. Further research and standardization approaches are essential to completely leverage the predictive power of these biomarkers and integrate them effectively into clinical practice for cancer immunotherapy.

**Table 4:** Ongoing clinical trials of TIME-related signatures and HLA to predict immunotherapy in recent years.

Biomarker	Clinical trial identifier	Phase	Number of patients	Cancer type	Treatment	URL
CD8+ TILs	NCT05270824	phase 3	120	Advanced gastric carcinoma	Neoadjuvant immunotherapy	https://clinicaltrials.gov/study/NCT05270824
Percentage of CD4+CD25+ CD127low FOXP3+ Treg cells	NCT03628859	/	30	Renal cell carcinoma	Nivolumab	https://clinicaltrials.gov/study/NCT01884168
CD8+/CD4+ TILs	NCT03267836	phase 1	9	Recurrent radiation-refractory meningioma	Neoadjuvant avelumab + hypofractionated proton therapy	https://clinicaltrials.gov/study/NCT03267836
levels of CD3+, CD4+, CD8+ lymphocytes	NCT03447678	phase 2	65	Stage IIIB-IV, PD-L1 low non-small cell lung cancer	Pembrolizumab	https://clinicaltrials.gov/study/NCT03447678
TILs, CD8+ lymphocytes	NCT05088889	phase 1	10	Stage IV pancreatic cancer	Radiation + ipilimumab + nivolumab	https://clinicaltrials.gov/study/NCT05088889
CD8+ T lymphocytes	NCT02554812	phase 1b/2	409	Locally advanced or metastatic solid tumors	Avelumab + other cancer immunotherapies	https://clinicaltrials.gov/study/NCT02554812
CD3, CCD4, D4/CD8 ratio, FOXP3	NCT04238988	phase 2	45	Advanced cervical cancer	Pembrolizumab + chemotherapy	https://clinicaltrials.gov/study/NCT04238988
Tregs, CD8 TILs	NCT03602586	phase 2	14	Ovarian clear cell carcinoma	Pembrolizumab + epacadostat	https://clinicaltrials.gov/study/NCT03602586
Immune cell infiltrates of IGHM, CD3, CD8, FOXP3, CD68, CD205	NCT04522544	phase 2	55	Hepatocellular carcinoma	Y-90 SIRT + tremelimumab + durvalumab or DEB-TACE + tremelimumab + durvalumab	https://clinicaltrials.gov/study/NCT04522544
TIS	NCT05136196	phase 2	150	Advanced melanoma or squamous cell head and neck cancer	Cabozantinib + nivolumab	https://clinicaltrials.gov/study/NCT05136196
HLA class I	NCT03602586	phase 2	14	Ovarian clear cell carcinoma	Pembrolizumab + epacadostat	https://clinicaltrials.gov/study/NCT03602586
HLA	NCT04636047	/	450	Non-small cell lung cancer	Immune checkpoint inhibitors	https://clinicaltrials.gov/study/NCT04636047

TIME, tumor immune microenvironment; TILs, tumor infiltrating lymphocytes; TIS, tumor inflammation signature; HLA, human leukocyte antigen.

### 3.2 Human leukocyte antigen

Human leukocyte antigen (HLA) molecules, also referred to as the major histocompatibility complex in humans, play an indispensable role in the immune system in presenting peptide antigens to T cells. HLA genes reside on the short arm of human chromosome 6 and there are three classes of HLA molecules: HLA-I (HLA-A, HLA-B, and HLA-C), HLA-II, and HLA-III (69). HLA molecules are highly polymorphic, with thousands of alleles identified, allowing for diverse peptide presentation and immune recognition (70). In the realm of cancer immunotherapy, HLA molecules are integral for presenting tumor-specific antigens to T cells, thereby triggering antitumor immune responses. Tumor-specific antigens, derived from mutated or aberrantly expressed proteins in cancer cells, are processed and presented by HLA molecules on the surface of antigen-presenting cells or tumor cells themselves. Recognizing these antigens, cytotoxic T lymphocytes will be activated and then directly target and eliminate cancer cells. Consequently, patients with higher HLA diversity or heterozygosity are often correlated with better response rates and overall survival in checkpoint inhibitor cancer immunotherapy.

The study by Chowell et al. (2018) demonstrated that the heterogeneity of HLA-I was associated with enhanced OS in patients treated with ICIs (71). They retrospectively analyzed high-resolution HLA class I genotyping in 1535 advanced cancer patients undergoing ICI-based immunotherapy. The study divided participants into two cohorts based on cancer type. The maximal heterozygosity at HLA-I locus was associated with increased OS in Cohort 1 (HR = 1.4, 95% CI 1.02-1.9, p = 0.036), Cohort 2 (HR = 1.31, 95% CI 1.03-1.7, p = 0.028), and the integration of two cohorts (HR = 1.38, 95% CI 1.11-1.7, p = 0.003) compared with homozygosity of at least one locus. Additionally, the HLA-B44 subtype was associated with extended survival (HR = 0.61, 95% CI 0.42-0.89, p = 0.01), while the HLA-B62 subtype corresponded to shorter survival times (HR = 2.29, 95% CI 1.4-3.74, p = 0.0007). In a different study designed by Rodig et al. (2018), patients from two previously published clinical trials, CheckMate 064 and CheckMate 069, were enrolled, where pre-treated metastatic melanoma patients received either monotherapy or combination therapy of ipilimumab and nivolumab (72). A correlation between reduced expression of HLA class I molecules (≤50% of cells) and worse OS was observed in patients treated with single agent ipilimumab in both the CheckMate 064 cohort (HR = 0.38, 95% CI 0.18-0.82, p = 0.01) and CheckMate 069 cohort (HR = 0.34, 95% CI 0.11-1.03, p = 0.057). Another study proposed the use of the HLA-I evolutionary divergence (HED) score to measure how the HLA-I diversity predicts the efficacy of ICIs (73). Patients were categorized into three cohorts based on cancer type and treatment regimen. It was observed that patients exhibiting a high mean HED score (mean HED level exceeding the upper quantile) had longer OS than those with a low score across each of the three cohorts and the combined cohort. Furthermore, a significant correlation was noted between a high mean HED score and an improved response to ICIs within the combined cohort. Several ongoing clinical trials also provide clinical evidence supporting HLA as a potential and valuable predictive biomarker for ICIs (**Table 4**).

The aforementioned evidence affirms the significant role that HLA, particularly HLA-I, plays in the efficacy prediction of immune checkpoint inhibitors. However, the HLA system is highly polymorphic, and its complexity can pose challenges in standardizing measurements and interpretations. Variability in HLA typing methods can also affect the reliability of the results. Intra-tumoral heterogeneity can affect the presentation of neoantigens, and despite high HLA diversity, some tumor regions may not present immunogenic neoantigens effectively, leading to variable responses to ICIs. In addition, factors such as the tumor microenvironment, presence of immune-suppressive cells, and the overall immune status of the patient, can influence the efficacy of ICIs and may confound the predictive value of HLA diversity alone. There may be additional mechanisms beyond broader neoantigen presentation through which HLA diversity may influence ICI efficacy, which are not fully understood. Therefore, continued research efforts on how HLA diversity or specific subtypes could benefit patient selection and optimize treatment strategy selection in cancer immunotherapy of various cancer types are needed. The mechanisms of how these confounding factors affect the HLA in predicting the ICIs efficacy should also be answered.

### 3.3 Non-invasive derived biomarkers

The aforementioned biomarkers, related to the TIME, are derived from tumor tissue biopsies obtained through invasive procedures such as puncture or surgery. Nevertheless, the tissue-based assay is encumbered by several limitations. The process of acquiring tissue samples is invasive, potentially harmful to patients, and incapable of tracking the dynamic change over time. Furthermore, due to intratumoral heterogeneity, tissue specimens from a single site may not accurately reflect the overall tumor characteristics of the patient (74). Consequently, blood sample-based detection methods have been developed to address these issues, providing a non-invasive and dynamic monitoring approach that is more acceptable to patients (75). Circulating tumor DNA (ctDNA) are the DNA fragments that tumor cells release into the blood or other body fluids (76). ctDNA can be used to detect early-stage cancers, predict treatment effectiveness, guide treatment strategy selection, detect minimal residual disease, and offer dynamic monitoring during treatment (77, 78). The abundance of ctDNA itself, the blood-based TMB (bTMB), and blood-based MSI (bMSI) derived from the ctDNA could serve as potential efficacious biomarkers associated with the therapeutic response to cancer immunotherapy (**Table 5**).

**Table 5:** Clinical trials of non-invasive biomarkers to predict immunotherapy in recent years.

Biomarker	Trial	Cancer type	Number of patients	Immunotherapy strategy	Clinical end points	Main results	Reference
ctDNA	INSPIRE (NCT02644369) phase 2	5 advanced solid tumors	94	Pembrolizumab	ORR, PFS, OS, CBR	Patients with baseline ctDNA level below the median had better ORR, PFS, OS, and CBR. Negative ΔctDNA^a^ was associated with favorable ORR, PFS, OS and CBR.	PMID: 35121950 (79)
ctDNA	POLARIS-03 (NCT03113266) phase 2	Metastatic urothelial carcinoma	27	Toripalimab	ORR, PFS, OS	Early ctDNA response and dynamic ctDNA changes were associated with toripalimab effectiveness.	PMID: 37584165 (132)
ctDNA	IMvigor010 (NCT02450331) phase 3	Muscle-invasive urothelial carcinoma	581	Adjuvant atezolizumab or observation	DFS, OS	Patients with positive ctDNA status showed much longer OS with atezolizumab than observation (p < 0.001).	PMID: 37500339 (133)
ctDNA	ABACUS (NCT02662309) phase 2	Muscle-invasive urothelial cancer	95	Neoadjuvant atezolizumab	pCR, RFS, DFS, OS	ctDNA positive patients exhibited a much higher relapse rate than ctDNA negative patients (PFS, p < 0.001)	PMID: 35577646 (92)
ctDNA	BISCAY (NCT02546661) phase	Advanced urothelial cancer	391	Durvalumab + targeted therapies	PFS, OS	Baseline ctDNA level above the median was associated with shorter OS.	PMID: 33941921 (134)
bTMB	B-F1RST (NCT02848651) phase 2	Advanced or metastatic non-small cell lung cancer	152	Atezolizumab	ORR, PFS, OS	ORR in the bTMB-H (≥16 muts/Mb) patients was significantly higher than the bTMB-L patients (35.7% vs 5.5%; p < 0.0001). bTMB-H was significantly associated with longer OS (p = 0.032).	PMID: 35422531 (81)
bTMB	BFAST cohort C (NCT03178552) phase 3	Advanced or metastatic non-small cell lung cancer	472	Atezolizumab or chemotherapy	ORR, PFS, OS	Atezolizumab-treated patients with a bTMB ≥ 13.6 muts/Mb had improved PFS than the chemotherapy-treated patients (p = 0.029).	PMID: 35995953 (83)
bTMB	(NCT03855358) phase Ib	Advanced triple-negative breast cancer	34	Benmelstobart + anlotinib	ORR, PFS, OS	Patients with bTMB-L (< 6.7 muts/Mb, median level) showed better ORR (p = 0.015) and longer PFS (p = 0.012) than those with bTMB-H (≥ 6.7 muts/Mb).	PMID: 37973901 (85)
bTMB	CO.26 Study (NCT02870920) phase 2	Advanced colorectal cancer	180	Durvalumab + tremelimumab or BSC	OS	Patients with high bTMB level (≥ 28 muts/Mb) had significant OS benefit with durvalumab plus tremelimumab than BSC (p = 0.004)	PMID: 32379280 (135)
bTMB	MYSTIC (NCT02453282) phase 3	Metastatic non-small cell lung cancer	1118	Durvalumab or durvalumab + tremelimumab, or chemotherapy	ORR, PFS, OS	Patients with a bTMB ≥ 20 muts/Mb had better median OS, 24-month OS, PFS, and ORR for durvalumab plus tremelimumab than chemotherapy.	PMID: 32271377 (43)
bMSI	CO.26 Study (NCT02870920) phase 2	Advanced colorectal cancer	180	Durvalumab + tremelimumab or BSC	OS	In bMSS patients/subgroup, OS was significantly improved with durvalumab + tremelimumab than BSC (p = 0.02).	PMID: 32379280 (135)

a : ΔctDNA, a lower ctDNA level at the beginning of 3 cycles pembrolizumab treatment versus baseline; ctDNA, circulating tumor DNA; bTMB, blood-based tumor mutational burden; bMSI, blood-based microsatellite instability; bMSS, blood-based microsatellite stability; ORR, objective response rate; PFS, progression-free survival; OS, overall survival; CBR, clinical benefit rate; DFS, disease-free survival; pCR, pathological complete response; RFS, relapse-free survival; BSC, best supportive care.

#### 3.3.1 Circulating-tumor DNA

The INSPIRE (NCT02644369) prospective trial evaluated ctDNA as a predictive biomarker for pembrolizumab monotherapy across five advanced solid tumors (79). ctDNA levels were detected at the baseline and after every three cycles of pembrolizumab. Patients with baseline ctDNA level below the median had better ORR (OR = 3.24, 95% CI 1.19-8.8), OS (adjusted hazard ratio (aHR) = 0.49, 95% CI 0.29-0.83), and PFS (aHR = 0.54, 95% CI 0.34-0.85). The trial also showed that the decreased ctDNA level after three cycles of pembrolizumab indicated a positive response to ICIs and a favorable prognosis. A retrospective study of advanced melanoma also revealed that the elevated ctDNA level after six weeks of ICI therapy compared to the pre-treatment level was significantly associated with worse PFS (HR = 22, p = 0.006) (80). Changes in ctDNA level associated with ICI efficacy were also validated in a large cohort integrated 18 trials of advanced solid tumors. Al-Showbaki L et al. (2023) analyzed published clinical trials with ICI administration and multi-timepoint ctDNA level screening (including both pre-treatment and on-treatment) (76). For all cancer types of the integrated cohort, patients with diminished or undetected ctDNA levels were significantly linked to an elongated PFS (HR = 0.20, 95% CI 0.14-0.28, p < 0.001) and OS (HR = 0.18, 95% CI 0.12-0.26, p < 0.001).

#### 3.3.2 Blood-based tumor mutational burden

In the prospective phase 2 B-F1RST trial, 119 NSCLC first-line atezolizumab-treated patients were accessed for bTMB levels (81). Using the threshold from the POPLAR and OAK trials, ORR in the bTMB-H (bTMB ≥ 16 muts/Mb) patients was significantly higher than that in the bTMB-L patients (35.7% vs 5.5%, p < 0.0001) (82). When the median follow-up reached 36.5 months, bTMB-H was significantly associated with longer OS (HR = 0.54, 90% CI 0.34-0.87, P = 0.032). Cohort C of the BFAST study of NSCLC also found a distinguishable cutoff of FoundationOne Liquid Companion Diagnostic detected bTMB ≥ 13.6 muts/Mb, with improved PFS observed in the atezolizumab-treated patients than the chemotherapy-treated patients (p = 0.029) (83). It was observed that advanced solid tumor patients with bTMB-H (bTMB ≥ 14 muts/Mb) had significantly better ORR than other patients with TMB-L in the SCRUM-Japan MONSTAR-SCREEN cohort (p = 0.05) (84). However, a phase Ib trial of 31 advanced triple-negative breast cancer patients treated with combination therapy showed that low-bTMB level (bTMB < 6.7 muts/Mb) was significantly associated with better ORR and PFS (p = 0.015 and p = 0.012, respectively), indicating that the efficacy of bTMB as a biomarker may vary by cancer type (85).

#### 3.3.3 Blood-based microsatellite instability

bMSI was found to be a significant predictor of both PFS (HR = 0.15, p = 0.001) and OS (HR = 0.26, p = 0.01) for pembrolizumab-treated metastatic tumors when the patients had adequate ctDNA (86). Wang, et al. (2020) designed a retrospective study comprising 60 patients diagnosed with advanced gastrointestinal (GI) cancer and treated with anti-PD-(L)1 immunotherapy (87). bMSI was detected using a targeted panel of 150 genes through blood samples. In this study, a better ORR (38.71% vs 6.90%, p = 0.005), and prolonged PFS (HR = 0.431, 95% CI 0.236-0.787, p = 0.005) and OS (HR = 0.489, 95% CI 0.249-0.961, p = 0.034) were observed in patients with bMSI-H compared with patients with bMSS. In another study of advanced GI cancer, the Guardant360 assay was utilized to obtain the MSI status through the ctDNA of patients before receiving ICI therapy (88). It garnered a similar outcome that MSI-H patients treated with ICIs showed significantly prolonged real-world time to discontinuation and real-world time to the next treatment (similar to PFS) than those treated with chemotherapy or other treatments (p < 0.001 and p = 0.006, respectively).

ctDNA in the blood is a promising source of biological biopsies, and it facilitates the early diagnosis and dynamic monitoring of disease in a non-invasive and more acceptable manner (89). Zulato et al. (2022) explored how the longitudinal cell-free DNA (cfDNA) can predict the hyperprogression and early death of ICI-treated advanced NSCLC patients (90). Plasma samples were collected at 2 time points: baseline (T1), and after 3/4 weeks of ICI (T2). Significant correlations between cfDNA levels at both T2 and the change between the 2 time points and high risk of early disease were observed. In a prospective study of advanced NSCLC, liquid biopsies of 113 patients at baseline (before treatment, T1) and after 2 or 3 weeks of ICI (T2) were collected (91). Patients with higher (median value cutoff) cfDNA at T1 and T2, and elevated cfDNA compared to baseline (ΔT2-T1) had significantly worse survival (both PFS and OS) and high progression risk.

In addition to predicting the treatment outcomes, ctDNA can predict the relapse as well. In the ABACUS (NCT02662309) trial of phase 2 muscle-invasive urothelial cancer, ctDNA positive patients were more likely to relapse compared to ctDNA negative patients (p < 0.001) (92). Similar results on muscle-invasive urothelial carcinoma were observed in the phase 3 IMvigor010 (NCT02450331) trial, in which patients with positive ctDNA showed higher relapse ratio after 3 cycles of treatment (p < 0.0001) (93).

However, the low levels of ctDNA in the blood have become the major challenge with blood-based liquid biopsies, which may result in reduced sensitivity for ctDNA detection, especially in the early stage of the disease (94, 95). A pan-cancer research enrolled more than 10,000 Chinese patients reported the ctDNA detection rate among different cancer types, in which most of the stage IV disease had detectable levels (79.7%), while the detection rate of stage I-III disease was just 57.9% (96). Another unsolved issue is that the degree of defining ctDNA decrement is uncertain. Therefore, more sensitive methods should be developed to increase the detection rate of ctDNA in the peripheral blood, and a standard of decrement of the change and detection time needs to be further constructed.

### 3.4 Multivariable predictive models

The biomarkers described previously are independent predictive biomarkers, and their individual correlation to ICI efficacy may be somewhat limited. To address this, the multi-biomarker model, which was developed using multiple such biomarkers or by integrating multi-omics data, facilitates the creation of more robust and powerful biomarkers. These models can more precisely and comprehensively predict the effectiveness of ICI immunotherapy by integrating the attributes of multiple biomarkers or multiple characteristics of patients.

#### 3.4.1 DIREct-On model

The DIREct-On (Durable Immunotherapy Response Estimation by immune profiling and ctDNA) model incorporates three non-invasive biomarkers, pre-treatment ctDNA-normalized bTMB, circulating CD8 T cell fraction and on-treatment early ctDNA dynamics, and it was initially constructed to predict the clinical response of NSCLC patients who received anti-PD-(L)1 immunotherapy (97). DIREct-On is a robust binary classifier for the clinical responses of patients undergoing PD-1/PD-L1 inhibitors, classifying patients into durable clinical benefit and no durable benefit. Patients exhibiting high DIREct-On scores demonstrated markedly prolonged PFS in comparison to those with low DIREct-On scores in both training and validation cohorts (HR = 8.93, p < 0.0001 and HR = 7.11, p < 0.0001, respectively). Meanwhile, it is also validated that the DIREct-On model significantly outperforms each of the individual ones, and each of the three features is indispensable for the optimal performance model. DIREct-On was also confirmed to be an independent predictor and the only factor significantly associated with PFS (HR = 0.06, p < 0.0001) in the multivariable Cox proportional hazard model of DIREct-On score, age, ECOG performance status, and line of therapy. The classification accuracy of the DIREct-On model for patients with different cancer types and different treatment modalities requires confirmation through larger cohorts of prospective studies.

#### 3.4.2 RF16 model

RF16 is a random forest-based model established to predict the potential of patients responding to immunotherapy (98). The model incorporates a total of 16 genomic features and clinical and demographic features. A total of 1,479 samples from the MSK-IMPACT cohort were enrolled, and they were randomly divided into the training set and the testing set at a ratio of 4:1. Those predicted as responders by the RF16 model showed significantly longer OS compared to the non-responders in both the training set (HR = 0.31, 95% CI 0.26-0.36, p < 0.0001) and testing set (HR = 0.29, 95% CI 0.21-0.41, p < 0.0001). Additionally, significantly longer PFS was also observed among the responders. The RF16 model achieved the best predictive performance in separating patients into responders and non-responders compared with each of the individual covariant.

#### 3.4.3 A XGBoost multivariable model

A meta-analysis including over 1,000 ICI-treated patients across seven types of cancers from several published studies constructed a multivariable model employing the XGBoost machine learning algorithm (99, 100). It comprised 11 features related to the host, tumor, and TIME. This multivariable model classifies the patients into responders and non-responders according to the RECIST criteria. In order to evaluate the predictive capability of the multivariable model, the FDA-approved TMB was used as a benchmark for comparison. The performance of the model was tested in three independent cohorts, and the multivariable predictive model significantly outperformed the TMB level in predictive accuracy. For example, the data from validation cohort 1 was from the KEYNOTE-28 trial, and the AUC (area under the curve) value of the XGBoost multivariable classifier was significantly higher than that of the TMB (AUC 0.86 vs 0.68, p = 0.0049) (101). Consistent results were obtained from the additional two test cohorts (p = 0.025 for cohort 2 and p = 0.047 for cohort 3).

#### 3.4.4 LORIS model

Chang et al. (2024) constructed a pan-cancer predictive model referred to as LORIS (logistic regression-based immunotherapy-response score), which was developed using six clinical and tumor-related features using retrospectively collected data from ICI-treated samples from MSK cohort and other six published cohorts, and non-ICI-treated samples from MSK cohort (102). The six-feature (TMB, systematic therapy history, blood albumin level, blood neutrophil-lymphocyte ratio, age, and cancer type) classifier for beneficiary patients of ICIs was constructed using logistic regression. LORIS outperformed other machine learning-based models, as well as the aforementioned RF16 model constructed by Chowell et al. (2022) (98). It was observed that high LORIS was associated with prolonged PFS (HR = 3.2, 95% CI 2.6-3.9, p < 0.001) and OS (HR = 2.6, 95% CI 2.2-3.0, p < 0.001). Additionally, LORIS outperformed independent TMB and can identify low-TMB patients who would like to benefit from ICIs.

### 3.5 Other biomarkers

In spite of the biomarkers mentioned above, other biomarkers like the gut microbiome, emotional stress, and images of histology slides or CT can also indicate the response to checkpoint inhibitors.

#### 3.5.1 Gut microbiome

The gut microbiome, which encompasses the entire collection of microbiotas (bacteria, the Archaea, viruses, and fungi), as well as their genes and products, such as metabolites, within both the lumen and mucosa of the gastrointestinal tract, has recently emerged as a pivotal factor influencing the efficacy of immunotherapy (103–105). A study revealed that patients with a more diverse gut microbiome prior to anti-PD-1 therapy demonstrated enhanced responses (106). It also showed that enrichment of specific strains of gut microbiota, such as *Akkermansia muciniphila* was significantly enriched in patients with better PFS. A machine-learning constructed signature quantifying 22 gut microbial strains was able to distinguish responders from non-responders in pan-cancer cohorts (107).Based on these studies, it can be concluded that changing the composition or abundance of gut microbes may also effectively enhance the anti-cancer efficacy, and improve the survival of patients.

#### 3.5.2 Psycho-biomarkers

In addition to the technically detectable features, psychological factors (psycho-biomarkers) may also influence the response to ICIs (108). Emotional distress (ED) refers to the adverse emotional states or feelings triggered by stressful stimuli, which is typically assessed using questionnaire-based surveys, and it is prevalent in cancer patients (after being informed of cancer diagnosis) (109). Zeng et al. (2024) designed a prospective study using the STRESS-LUNG-1 NSCLC cohort treated with various kinds of anti-PD-1/PD-L1 inhibitors, and found that patients with baseline ED (high ED and moderate ED) had significantly shorter DFS, OS, and smaller ORR compared with no ED patients (108). The result of this study implies that addressing the ED could be a potential approach to improve the efficacy of ICIs. In future research, the reliability of ED should be validated in larger cohorts with pan-cancer. It also provides an inspiration that other confounding factors may also influence the ICIs’ efficacy.

#### 3.5.3 Image-derived biomarkers

Images of histology slides or the CT images also have potential in predictive model construction of immunotherapy response. Johannet et al. (2021) constructed a deep convolutional neural networks-based model, which extracted features from the whole slide images of melanoma patients, to stratify the patients into the high and low risk of progression (110). The patients with low progression risk were validated to have significantly longer PFS than the high risks in two validation datasets. Huang et al. (2023) developed a CT imaging biomarker of pretreatment samples to predict the response of patients who underwent anti-PD-1/PD-L1 immunotherapy (111). Features selected from each CT image were conducted to a radiomics score (RS), and the patients were divided into the RS-high and RS-low groups (threshold 0.22). Compared to patients with RS-high, RS-low patients showed significantly lower rate of progression disease and longer median PFS, 12-month PFS, and 12-month OS.

## 4 Combination biomarker strategies

To enhance the predictive accuracy of immunotherapy responses, the combination of two biomarkers has emerged as a promising strategy, especially in cases where individual biomarkers have shown limited predictive power.

### 4.1 PD-L1 expression and TMB

Enhanced predictive accuracy of immunotherapy can be achieved by combining the two FDA-approved biomarkers, PD-L1 expression and TMB, rather than using them independently (112). In the CheckMate-016 trial of NSCLC, nivolumab-treated patients displaying both elevated TMB levels and PD-L1 expression above 50% demonstrated an increased response rate compared to patients with only one of these features (113). Similar findings were observed in the CheckMate 275 trial of nivolumab-treated urothelial carcinoma. Despite an absence of correlation between PD-L1 expression and TMB in this study, the combination of these two biomarkers outperformed PD-L1 alone in predicting PFS and OS (p = 0.0056 and p = 0.013, respectively) (24). Notably, results from CheckMate 568 trial on NSCLC demonstrated no association between PD-L1 expression and TMB. No matter the PD-L1 expression levels, patients with high TMB levels showed higher ORR and longer PFS compared to those with low TMB levels (114).

### 4.2 PD-L1 expression and TILs

The combination of PD-L1 and tumor infiltrating lymphocytes also indicates the effectiveness of ICIs. In a study of metastatic melanoma, PD-L1+/CD8+TILs (PD-L1 expression on tumor cells (TC) ≥ 5%) status was identified as a significant independent prognostic factor for improved OS (HR = 0.138, 95% CI 0.024-0.779, p = 0.022) (15). A study of NSCLC also elicited analogous findings. Patients with high CD8+ PD-L1+ TILs levels had longer PFS than patients in the low-level group (HR = 0.55, p = 0.0429), where PD-L1 positivity was defined as ≥ 1% of TC staining (115).

### 4.3 HLA class I and other biomarkers

Improved predictive efficacy of HLA class I was noted when combined with other biomarkers. In the NTR7015 study of 30 nivolumab-treated NSCLC patients, correlations between combinations of HLA and TMB, CD8+ T cell infiltration, and PD-L1 expression with survival were observed (116). The combination of no loss of HLA class I and (1) high TMB (p = 0.023) (2), CD8+ T cell infiltration (p = 0.041) (3), high PD-L1 expression (p = 0.032) was correlated with better PFS. The research of Montesion et al. (2021) indicated the combination of HLA class I loss of heterozygosity (LOH) and TMB as more powerful biomarker of ICIs (117). Patients with HLA-I LOH and TMB-L (TMB < 10 muts/Mb) had the worst PFS, and the predictive efficacy was improve compared to individual biomarker.

## 5 Discussion and future perspective

Immunotherapy, particularly ICIs, has emerged as a revolutionary approach in the cancer treatment landscape. The ability of ICIs to harness the immune system of patients to target and eradicate cancer cells has led to significant advancements in the management of various malignancies. However, the response to ICI therapy is highly variable, with only a subset of patients experiencing durable clinical benefits. This variability underscores the urgent need for reliable biomarkers to predict treatment outcomes and to guide personalized treatment strategies.

The current review has extensively discussed the role of biomarkers in predicting the efficacy of ICIs. The three FDA-approved biomarkers, PD-L1 expression, TMB, and MSI, have shown promise but with limitations. PD-L1 expression, while a valuable predictor, is not uniformly predictive across all cancer types and can be influenced by various factors such as variations in detection platform, quality of tumor specimens, tumor heterogeneity and dynamic changes during the treatment. TMB, though associated with clinical benefits, faces challenges in standardization across different sequencing platforms and cancer types, selection for optimal representative sample, and the impact of specific genetic ethnicity. MSI, identified as a predictor of response primarily in endometrial cancer, colorectal cancer, and gastric cancer, has limited predictive power in other cancer types.

The search for more robust and accurate biomarkers has led to the exploration of other potential markers, such as TILs, HLA diversity, ctDNA, gut microbiome, emotional distress, and image-derived biomarker. TILs have been shown to correlate with improved responses to ICIs, but challenges remain in standardizing assessment methods and interpreting their presence in the context of complex tumor microenvironments. HLA plays a crucial role in antigen presentation, and its polymorphism has been linked to improved outcomes in ICI therapy. However, the influence of specific HLA genotypes or the expression level on treatment response requires further elucidation. ctDNA, offering a non-invasive approach to assess tumor characteristics, has emerged as a promising biomarker as well. It holds significant promise for revolutionizing cancer management by enabling early disease detection, monitoring treatment efficacy, and predicting relapse. However, challenges related to the sensitivity of detection rate, and standardization must be addressed. The mechanism of gut microbiome demonstrating treatment outcome is unclear, since in addition to the diversity and enrichment of themselves, they are also greatly affected by the diet and environment of the host. Emotional distress is technically undetectable and more subjective, which poses a great challenge to clinical applications. Image-derived biomarker indicates the features extracted from histological or CT images, but it relies heavily on the deep learning models and large dataset for model construction.

The integration of multiple biomarkers into multivariable predictive models presents a promising strategy to improve predictive accuracy. The DIREct-On score, RF16, XGBoost multivariable model, and LORIS model are examples of such approaches, combining various features and multi-omics data to discriminate immunotherapy responsive patients. These models have demonstrated improved predictive capabilities over individual biomarkers, suggesting that an integration of biomarkers may provide a more comprehensive assessment of a patient’s likelihood to respond to ICIs. However, standardized and integration of these multivariable predictive models into clinical practice constitute a significant challenge. These models first need to be replicated in other independent validation cohorts, and secondly, sufficiently large datasets are needed to retrain and test the model to achieve the optimal predictive effects. It is important to note that the integration of these models into clinical practice should be guided by evidence from well-conducted clinical trials.

Despite the progress in biomarker research, several challenges remain. The intra- and inter-heterogeneity of tumors, differences in immune microenvironments, and the influence of various genetic and non-genetic factors complicate the development of universal biomarkers. Furthermore, the dynamic nature of the tumor-immune interaction implies that the expression of biomarkers may change over time, necessitating longitudinal assessments.

Looking ahead, the future of biomarker research in immunotherapy lies in several directions. First, the standardization of biomarker assessment across different platforms and studies is crucial to ensure consistency and reproducibility of results. Second, the development of more sensitive and specific assays for biomarker detection, particularly for ctDNA, will enhance the clinical utility of these markers. Third, the exploration of novel biomarkers, including those derived from the immune microenvironment and the tumor’s metabolic profile, may uncover new avenues for predicting treatment response.

The integration of multi-omics data, genomics, transcriptomics, proteomics, metabolomics, and clinical data, may provide a more holistic view of the tumor-immune dynamics and improve predictive models. Machine learning and artificial intelligence can aid in deciphering complex patterns and interactions between various biomarkers, potentially identifying synergistic combinations that predict treatment outcomes more accurately. Moreover, the role of non-molecular factors, such as psychological stress, in modulating treatment response to ICIs warrants investigation. The influence of the patient’s psychological state on their immune system and, consequently, on the efficacy of immunotherapy is an emerging area of research that may lead to novel psycho-biomarker discovery.

In conclusion, the discovery and validation of effective biomarkers for ICI therapy are critical for advancing personalized cancer treatment. While significant strides have been made, the journey towards precision immunotherapy is ongoing. Future research should focus on addressing the current limitations, standardizing biomarker assessment, and exploring innovative approaches to biomarker discovery. By doing so, we can enhance the ability to predict and optimize treatment outcomes, ultimately improving patient care and quality of life in the era of immunotherapy.
